# Perspectives From Students and Faculty on How Women Achieve Leadership Roles in Academic Medicine: An Exploratory Qualitative Study

**DOI:** 10.7759/cureus.57969

**Published:** 2024-04-10

**Authors:** Candace J Chow, Meganne N Ferrel, Emily M Graham, Megan L Fix

**Affiliations:** 1 Office of Curriculum/Internal Medicine, University of Utah, Salt Lake City, USA; 2 Cardiac Surgery, University of Michigan, Ann Arbor, USA; 3 Surgery, Section of Plastic and Reconstructive Surgery, University of Michigan, Ann Arbor, USA; 4 Emergency Medicine, University of Utah, Salt Lake City, USA

**Keywords:** medical education curriculum, academic medicine, professional mentor, gender inequities, women in leadership

## Abstract

Introduction: The glass ceiling in academic medicine has resulted in lower pay and fewer career advancement opportunities for women. Creating change relies on preparing early-career women for positions of leadership, but most leadership programs focus on faculty, not trainees. The present exploratory qualitative study investigates how to prepare women medical students to be leaders in academic medicine. Methods: Focus groups with medical students and faculty who identify as women were conducted at an academic medical center in the West. A total of 25 individuals (10 students and 15 faculty) participated. Recordings of focus groups were transcribed and coded using thematic analysis until saturation of themes was achieved. Findings: Codes were organized into three themes: obstacles, support systems, and self-presentation. Obstacles identified included the subthemes microaggressions, macroaggressions, a lack of female role models in leadership, and personal characteristics such as the ability to self-promote and remain resilient. Support systems included sponsorship, allyship, mentorship, networking, and gender-specific role modeling subthemes. Self-presentation involved learning behaviors for demonstrating leadership and exuding confidence, being strategic about career moves, resiliency, and navigating social norms. Conclusions: The key themes of obstacles, support systems, and self-presentation are targets for systemic and individualistic improvement in leadership development.

## Introduction

The glass ceiling, the proverbial barrier to career advancement, is a familiar concept to women in medicine who earn lower pay, have fewer career advancement opportunities, and hold fewer tenured faculty positions [1-5], despite the fact that more women than men are entering medicine [6]. While there are programs designed to help women circumvent these obstacles, most of these programs are designed to help women who are already in positions of leadership [7,8]. These programs, while needed, do not reach women at the beginning of their medical careers. Given that more women are starting to enroll in medical school than men, it is important to understand how to prepare early-career women, including students and trainees, to become leaders and break the glass ceiling.

Two of the authors on this team are former leaders of a grassroots women’s leadership group named WE WILL (Women Empowering Women in Leadership) at the Spencer Fox Eccles School of Medicine at the University of Utah (SFESOM). WE WILL aims to create space for and promote women leaders in medicine through networking events and skill-building workshops for women students and physicians that promote mentorship and professional development. Preliminary results from the WE WILL programming show success in empowering women for leadership positions after networking and skill workshop events (poster: Weaver M., Brecha, F., Sreekantaswamy, S., Neville, R., Nguyen, S., Fix, M.). WE WILL: Women Empowering Women in Leadership: A Model for Facilitating Successful Networking and Community Building. MWIA Centennial Meeting. (American Medical Women’s Association, 2019). Based upon WE WILL outcomes and greater university support, developing and implementing an elective leadership curriculum for students who identify as women will motivate more women to choose careers as academic leaders in medicine and better prepare them to succeed in those roles. Ultimately, by better-preparing women for positions of leadership within medicine, important perspectives and representation will be added to our healthcare system, along with enhanced patient care and decision-making processes [9-12].

This qualitative study was conducted to understand the challenges women medical students and faculty face in academic medicine and to inform the creation and implementation of a leadership curriculum for women medical students. The study, which also serves as a needs assessment, explored the support systems and resources participants have used to buffer these challenges. Findings from the study were previously presented as an oral presentation at the American College of Emergency Physicians (ACEP) Scientific Assembly Virtual Conference in October 2023. An earlier version of this manuscript was previously posted to the JAHSE: PRE preprint server on August 1, 2023.

## Materials and methods

This exploratory qualitative case study, which utilized purposive and maximum variation sampling, was deemed exempt by the University of Utah Institutional Review Board. Focus groups were conducted with University of Utah medical students and physician faculty who identified as women and who held positions of leadership. Student participants were recruited through an email invitation distributed to all students who were self-selected to participate after an open recruitment email was distributed to the school of medicine. One of the faculty authors (MF) assisted with identifying women faculty in positions of leadership to engage in purposive sampling of women leaders. Faculty were viewed as leaders if they had been promoted to at least the associate professor level or if they held a position that required them to lead a group of faculty, students, or curricular effort (e.g., chief, vice chair, program director, course director). Faculty were purposefully sampled to maximize diversity across medical specialties. As this study focused on women, only female-identifying medical students and faculty were asked to participate.

Our institution enrolls 125 students every year. In AY20-21, the year this study was conducted, only one of the four cohorts of students at our institution had more women than men (MS2021: 42%, MS2022: 49%, MS2023: 39%, MS2024: 54%). This same year, the percentage of faculty who identified as women was 42%, N=1663. Thus, this study explored the challenges women in academic medicine face in a context where women make up less than half of the student body and faculty population. 

Three student and three faculty focus groups were held. A total of 25 individuals participated (10 students and 15 faculty). Student focus groups were conducted by MNF and EMG, and faculty focus groups by MF. Participant consent was obtained by the individual conducting the focus group. Questions for the semi-structured focus groups were pilot-tested by the authors. Questions were designed to inquire about individual determination, aspiration, support, and obstacles to becoming a leader in medicine. Focus groups were conducted and analyzed until there was a saturation of themes [9]. To ensure focus group leaders understood participants correctly, accuracy was corroborated during the focus groups through reflexive feedback.

Focus groups were transcribed verbatim, and transcripts were coded and analyzed using thematic analysis [10]. The primary investigator reviewed all transcripts in detail and developed an initial codebook. Other authors then coded the transcripts. Disagreements were resolved by consensus, and revisions were made to the codebook. The authors (CJC, MNF, and EMG) then reviewed all transcripts to ensure the data were well-described by this revised codebook.

Positionality of researchers

The authors include one physician, one medical education researcher, and two medical students (both of whom are currently residents), all of whom identify as women. Having both faculty and students on the team afforded us rapport with the two participant groups of focus (faculty and students) and allowed us to explore faculty and student perceptions of the data.

## Results

Codes were organized into three themes: obstacles, support systems, and self-presentation. In the subsequent paragraphs, we describe these themes. Illustrative quotes are provided in Table 1.

**Table 1:** Themes, subthemes, and illustrative quotes We use ‘S’ to denote when a quote is from a student focus group and ‘F’ when a quote is from a faculty-student group. The number is used to distinguish between the student and faculty focus groups.

Theme	Subtheme	Quotes
Obstacles		
	Microaggressions	“when you're a petite female … I used to always get, ‘Hey nurse, am I going to see a doctor?’” (F4)
		“whatever we were juggling, I always contributed, I always worked hard or as hard, if not harder, I think, than a lot of people, and to have little barbs thrown at you because you were on maternity leave or because you went to your child's kindergarten…performance” (F3)
		“mind clutter, the worry you have to go through about ‘what am I going to wear today?’” (F4)
		“…we have a patient presentation and they just say, "Dress professionally." If you look around the room, the men just get to wear a button up shirt and slacks, and that is professional and it's easy. And the women have to figure out, ‘Is this dress long enough? Is this dress too low cut? Am I going to get looked at because my shoulders are out? Is a pencil skirt unprofessional?’ … These are things that are both internal and external barriers” (S2)
	Macroaggressions	“sometimes more overt comments about like ‘are we trying to hire a woman or are we trying to hire the best person for the job?’” (F3)
		“…I found out recently that there was a discussion that there should be a non-compete on my contract when I came here because I was going to get pregnant…but (it didn’t happen because)…somebody else had spoken up and said, "No, you can't do that, that's gender discrimination" (F3)
		“I have had one patient where I had to stop standing at a certain part of his bed because he could reach my leg and he would like grab and pat my leg…I just started standing on the far end of his bed where he couldn't reach it” (S2)
	Lack of women in leadership	“we still aren't seeing very many women leaders… there's systemic discrimination against women,” (S1)
		“I think this is an amazing institution, but … some of the things that frustrate me here are not indirectly related to (the fact that… department chair is) the only woman in that room where those decisions get made right now and that's just a problem” (F1).
	Lack of role models	“(I did not) have any physicians in (my) family” (S3)
		“no one in (my) family…is in academia” (F4)
		“growing up, I never saw a woman who's a minority in places that I wanted to be. I never saw a Latino doctor… I had so many questions growing up. Like ‘how do I even get to this place?’” (S1)
	Personal characteristics that need to be overcome	“… a man is going to ... take charge, and people will maybe listen to him more, and that's a fear in the back of my mind…it's definitely something I consider when I'm like, oh should I go after this position? Maybe it's not a good fit for me, and I think I tie that into being a female” (S3)
		“(My) biggest barrier has been myself. But going back to what (she) … said is, self-advocating. If you look at studies, women have been known to be, to undervalue themselves” (F2)
Support systems		
	Sponsorship	“The most important factor for me was a sponsor…He had just, he only spent two years in our department, but I happened to be his chief resident. And he sponsored me. I would have never had the job I had without him. I am 100 percent sure” (F2)
		“it was actually my immediate…supervisor within my division who saw my potential from my clinical interests to take over and was very great about…guiding me…into that role” (F3)
	Mentorship	“He also helped me say no to things and be like "You're too busy." So that was also, I think, a very important trait of a mentor, being interested and aware of everything that I was doing and to help me make the best choices" (S2)
	Allyship from men	“…(he) was actually really important for me because I didn't have female mentors because they didn't exist” (S2)
		“… it's kind of put me in this place where finding male mentors who recognize that it was harder to be a woman (is helpful)” (S2)
		“it's hard enough to have to go learn all these things. I do Step studying. I didn't do anything useful. I didn't do the dishes. I didn't my laundry. My husband did it all for me. ... I think it's huge” (S1)
	Gender-specific advice	“my mom would always say things like, "You're going to college," not like, "Are you going to college?...She was like, "…you're going to college, and you're doing it before you have kids. And you're not getting married before you graduate college’” (S2)
		“my mom was always the boss in my house, and she was always…the leader in any groups…And I would always see her running the show, and that was what I would look up to” (F3)
		“I think the most helpful thing for me has been to see the behavior modeled by my attendings and residents to show how they handle it. Because unfortunately, it happens to them on rounds in front of the entire team, just as much as it happens to me alone in an exam room before I go get the attending” (S2)
Self-presentation		
	Being strategic	“Find what motivates you and then go for it. Totally unabashedly. Go for it.” (F1)
		“embrace…knowing who you are and being authentic to who you are. Don't try on somebody else's thing. And there's not a type of person that is a leader; the effective people are the ones that know themselves well and are comfortable with being that person in that leadership space” (F3).
	Resilience	“roll with the punches instead of feeling flattened and demoralized” (F4)
		“I guess my own resilience and persistence has kind of kept me in the game. I'm also a little rebellious like, ‘You think I can't do this? Well, let me show you, I actually can’” (F3)
		“I think we faced all these different challenges, but as I reflect, I think they've made me stronger, more resilient. All these little battles that you have to fight. All these little obstacles on the road, and you kind of just keep plowing through” (F2)
		“looking back and see how far you've come can be a huge thing. Just remembering where you started and how hard it was just to get into medical school and how hard it was just to get through college…And just remembering that you can do hard things and you're totally capable” (S1)
	Navigating social norms	“I went through this process where I got rid (of it)… I only wear black, gray, and blue because then I only need black belts and black shoes” (F1)
		“I went to Singapore and I got a tuxedo made. And I wore tuxedos for events for a number of years” (F4)
		“pay other people to do all of that menial stuff for you, because you will be saner and calmer at the end of the day…If you were trying to do all the other stuff, you'd just be exhausted and frazzled and frustrated. (This way) you’re fulfilling your potential.” (F4)
	Advocating for other women	“Someone told me ‘This is going to be hard. There's no such thing as a good time for when you have kids. There's no such thing, … you just do it when you're ready. And that was really reassuring to me…" (F3)
		“…I had a …situation where incidentally, a male in a position of leadership said, ‘Don't ask for that. Don't ask for too much; just be grateful of what you're getting.’ And I went to some other women colleagues, and they said, ‘Ask for it; you need to make sure you get what you're worth and ask for these different opportunities in your leadership position,’ and I took the advice I wanted and asked for it.” (F3)
		“(women) have to identify and help each other and point out ways to improve and be better so that everybody can reach their maximum potential of where they want to be” (F2)

Obstacles

Participants reported various obstacles in their careers. These included microaggressions, macroaggressions, a lack of women in leadership, a lack of role models, and personal characteristics. Participants shared various microaggressions they had experienced as women. A common refrain was not being recognized as a physician. Participants shared how they were viewed differently because they were mothers. Several faculty and students commented on being critiqued about how they dressed or the fact that they needed to worry about how their clothes affected how they were perceived. Participants also shared macro-aggressions. Some reflected on biases exposed during hiring conversations. Others disclosed instances of sexual harassment.

The lack of women in leadership was a major concern shared among participants. Participants also mentioned how a lack of role models in their personal lives made their entry into medicine difficult. Others said they had a hard time picturing themselves in medicine because they had not seen themselves reflected in role models.

Participants also discussed how their own characteristics, which they had been socialized to adopt, got in the way of themselves. Students and faculty similarly reflected on how self-doubt could be detrimental to advancement.

Support systems

Participants spoke at length about support systems that helped them overcome the obstacles mentioned in the section above. Support included sponsorship, mentorship from colleagues, allyship from men in their lives, and gender-specific role modeling. Faculty recalled how important sponsorship was to their success. They shared that sponsors are not necessarily long-term mentors but are individuals who “put themselves on the line and give you an opportunity” (F3).

Participants also shared how mentorship played a key role in their success. Several faculty across focus groups reflected on how mentorship programs advanced their careers, like the Association of American Medical College’s (AAMC) Executive Leadership in Academic Medicine (ELAM) program and other career-advancement programs designed specifically for women [13]. Others explained that specific mentors helped pave their paths. Participants noted that “it wasn't necessarily people who looked like me or had the same path” (F1) that mentored them, but people who recognized their potential and provided encouragement. They shared that their network, through mentors and sponsors, was the key to opportunities.

More than one participant commented on how colleagues who are men were key to their success. Others spoke about the importance of having men who understood the discriminatory system as mentors. Participants also shared about the significance of having supportive life partners.

Finally, students and faculty spoke about gender-specific advice they had received. One shared about lessons she had learned from her mother, who did not go to college until she had children. Participants also shared about learning to respond to sexism from female role models.

Self-presentation

Participants (mainly the faculty) shared that another way to overcome obstacles was to learn how to act in ways that demonstrated leadership and/or exuded the confidence needed to succeed in academic medicine. These included learning to be strategic in their careers, being resilient, and navigating social norms. Participants also discussed how important it is for women to advocate for one another.

Participants shared how they strategized to get where they were. This included knowing what you wanted and learning to “gracefully self-promote” themselves (F4) to share their accomplishments. Others mentioned learning to be comfortable with themselves.

Resilience was also mentioned multiple times. Some felt being challenged was what helped them succeed. Students also said that reflecting on what they had accomplished was helpful.

Faculty shared how they learned to navigate social norms. In response to the discussion about constant criticism about how to dress, faculty discussed strategies for not having to make time-consuming decisions about what to wear. Faculty also shared that handling the “whole guilt thing” (F4) related to being a working mother involved prioritizing their children and their well-being.

Finally, participants spoke about the importance of women advocating for each other. One said that hearing about other women’s paths was helpful. Another said having female confidantes was key.

## Discussion

This qualitative study explored the challenges women medical students and faculty in academic medicine face. The study served as a needs assessment to inform the creation of a leadership curriculum for women medical students. Findings were organized into three themes: obstacles, support systems, and self-presentation, and each of the themes was further subdivided into sub-themes.

Participants explained that obstacles included implicit and explicit examples of gender discrimination, both of which are well documented in the literature [14-17]. Participants also noted that a lack of role models was another obstacle [18,19]. Finally, participants commented on personal characteristics they wished to change as obstacles. This is consistent with studies that show that imposter syndrome [20] is more likely to be experienced by women because they must constantly overcome societal norms and gender discrimination to be perceived as competent [21,22].

Participants mentioned benefiting from multiple types of support. Some recalled sponsors who helped them earn leadership opportunities or mentors who provided advice over time, which is associated with satisfaction [23] and women remaining in medicine [19]. Specifically, participants discussed how role-modeling specific to situations they might face as women was helpful. Others spoke about how men in their lives had supported them, which is important for women in medicine whose careers are less flexible than their spouses’ [24].

Faculty spoke at length about how they worked on self-presentation to advance their careers. Students did not speak much on this, but it is unclear whether it is because they have not had opportunities yet to demonstrate these skills or to hone them. Faculty discussed the importance of being strategic, spoke about navigating social norms, and emphasized the importance of women advocating for each other [25].

Together, these findings imply that current medical students who are women at SFESOM share many of the struggles that faculty who are women at SFESOM and across academic medicine have historically experienced. While we would expect microaggressions and macroaggressions towards women in academic medicine to lessen over time and the advancement of women to leadership positions to increase, it does not seem as though they have. Additionally, the solutions that participants spoke about finding supportive sponsors and role models, working on mitigating their imposter syndrome, advocating for one another, and even learning how to act so that their talents would be recognized for all solutions to singular problems at the individual level. None of these strategies address an inequitable system. For example, if women are not given the same opportunities as men, such as for networking and serving on hiring committees, this translates to fewer opportunities for career advancement [26]. The fact that lack of access to one opportunity affects access to additional opportunities reinforces how changes to gender inequities in medicine must be systemic rather than individual and that these systemic changes must be multi-pronged [27]. The study’s findings have provided many helpful suggestions for creating an institution-sanctioned curriculum to prepare women students for leadership, and such a curriculum would demonstrate a commitment to organizational change. However, women students also need to see this type of systemic commitment demonstrated in other ways and geared towards women in more senior positions at the postgraduate and faculty ranks.

Limitations

One study limitation is the inclusion of participants from a single center, which may differ from the experiences of women from other institutions and regions. However, since this study was ultimately performed to assist with the creation of a women's medical school leadership curriculum at SFESOM, the results are relevant to the study's investigators’ context and will likely be useful to others with similar goals. A second limitation is that the research questions and data analysis process investigated the role of gender alone and did not consider how participants’ experiences may be the product of intersectional identities. We acknowledge that the number of students and faculty who participated in the study is small relative to the total number of students and faculty at our institution. However, we were able to capture experiences from students and faculty in a context where women are not part of the majority. Additionally, a strength of qualitative projects is the descriptive data they generate, even from small sample sizes. 

## Conclusions

This exploratory qualitative study of medical students and faculty who identify as women used focus groups to learn about the challenges that women face in pursuing careers and leadership roles in academic medicine. Challenges identified included microaggressions, macroaggressions, a lack of women in leadership, and a lack of role models. Faculty participants also commented on support systems and how they learned to present themselves in ways that helped them fit into academic medicine.

Participants spoke largely about individual solutions to challenges, but mitigating obstacles on a systemic level is also required to address the glass ceiling for women in medicine. Creating more female leaders in academic medicine needs to be everyone’s responsibility, not just that of women. Moreover, rather than expecting women to accommodate the system, the rules of the system need to change.

## Appendices

Focus group questions

Introductory Questions

o Why did you decide to join our focus group today?

Questions on Resources

o What were helpful, contributing factors that helped shape your career (faculty) or education (students)?

o What outside training have you had in leadership development? (Example: Executive Leadership in Academic Medicine [ELAM] program)

o What advice do you have for others?

o What roles have mentors played in supporting/furthering your education/career? (students)

Questions on Intrinsic Characteristics

o What are essential skills a leader in medicine should develop in order to lead effectively?

o What helpful practices should medical students who are women or a minority gender do to develop leadership?

o What do you find are the intrinsic characteristics that may hinder success? What barriers to leadership have you experienced in your career?

Questions on Barriers: Let Participants Bring These Subjects Up Rather Than Leading Them to the Following Topics

o Have you experienced gender discrimination during your career? How?

o What is your experience with supporting staff and responsiveness to requests? (nurses, PAs, NPs, etc.).

o How do you feel that patients listen to you as a physician and a woman?

o What is your experience of male colleagues listening to you?

Questions to Encourage Follow-Up and Continuation of Ideas

o Do you disagree or agree with [name]’s statement, and why?

o [Name], you’ve been a bit quiet recently. Do you have any thoughts on this topic that you want to share?

o How did you learn that?
